# Evaluating the Antiviral Potential of Polyherbal Formulation (*Kabasura Kudineer*) Against Monkeypox Virus: Targeting E5, Poxin, and DNA Polymerase Through Multifaceted Drug Discovery Approaches

**DOI:** 10.3390/life15050771

**Published:** 2025-05-12

**Authors:** Sivan Padma Priya, Singamoorthy Amalraj, Vivek Padmanabhan, Mohammed Mustahsen Rahman, Nallan CSK Chaitanya, Nada Tawfig Hashim, Srinivasan Prabhu, Muniappan Ayyanar, Shailendra Gurav, Stanislaus Antony Ceasar, Rekha Thiruvengadam

**Affiliations:** 1 RAK College of Dental Sciences, RAK Medical and Health Sciences University, Ras Al Khaimah P.O. Box 12973, United Arab Emirates; vivek.padmanabhan@rakmhsu.ac.ae (V.P.); mustahsen@rakmhsu.ac.ae (M.M.R.); krishna.chytanya@rakmhsu.ac.ae (N.C.C.); nada.tawfig@rakmhsu.ac.ae (N.T.H.); 2 Division of Phytochemistry and Drug Design, Department of Biosciences, Rajagiri College of Social Sciences, Cochin 683 104, Kerala, India; s.amalraj101@gmail.com; 3 PG and Research Department of Botany, AVVM Sri Pushpam College (Autonomous) Poondi, Bharathidasan University, Thanjavur 613 503, Tamil Nadu, India; asmayyanar@yahoo.com; 4 Department of Pharmacognosy, Goa College of Pharmacy, Goa University, Panaji 403 001, Goa, India; shailendra.gurav@nic.in; 5 Division of Plant Molecular Biology and Biotechnology, Department of Biosciences, Rajagiri College of Social Sciences, Cochin 683 104, Kerala, India; antonysm2003@yahoo.co.in; 6 Department of Community Medicine, Saveetha Medical College and Hospitals, Saveetha Institute of Medical and Technical Sciences (SIMATS), Saveetha University, Thandalam, Chennai 602 105, Tamil Nadu, India; reklak4@gmail.com

**Keywords:** monkeypox, *Kabasura Kudineer*, MD simulations, DFT, toxicity

## Abstract

The recent reemergence of the monkeypox pandemic in non-endemic regions has raised serious concerns regarding the possibility of a global outbreak. The study employed various modules of the Schrodinger suite through Maestro V 14.1 for molecular docking, MD simulations, MM-GBSA, and FMO. To explore the drug potential of *Kabasura Kudineer* against the key proteins of the Mpox virus: E5, poxin, and DNA polymerase, a total of 982 chemical constituents belonging to this herbal formulation were investigated. The molecular docking studies revealed that chlorogenic acid, chebulic acid, rosmarinic acid, and citric acid had high binding affinities for E5, with docking scores of −13.3289, −11.3933, −9.8999, and −9.59471 kcal/mol, respectively. Likewise, caffeic acid, citric acid, and plumbagic acid have good binding affinities for poxin with docking scores of −8.49023, −6.80386 and −5.91719 kcal/mol, respectively. Plumbagic acid and delphinidin have considerable binding affinities for DNA polymerase with docking scores of −7.57867 and −7.55301 kcal/mol, respectively. In the MD simulation, chlorogenic acid, chebulic acid, citric acid, and rosmarinic acid exhibited remarkable stability with strong binding affinities for the E5, poxin and DNA polymerase. We further explored the stability of the E5 complexes by calculating the binding free energy every 20 ns for 100 ns. The ΔG bind values of chlorogenic acid, chebulic acid, and rosmarinic acid were 61.10, 78.14, and 75.49 kcal/mol at 0 ns. Hence, the research suggests that this formulation has antiviral potential against Monkeypox and can be used to inhibit viral replication in hosts and boost the antiviral immune response.

## 1. Introduction

**Figure 1:**
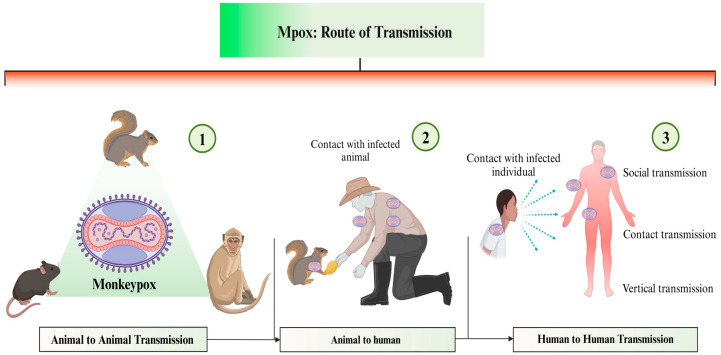
Transmission of Monkeypox virus (Created with www.BioRender.com).

In recent years, there has been an outbreak of various viral diseases worldwide, including Nipah virus, influenza virus (H1N1, H5N1), Ebola virus, Zika virus, and recently the coronavirus outbreak [1,2,3]. The most recent addition to this list is the monkeypox virus (MPXV of Mpox), one of the four human-pathogenic orthopoxvirus species alongside variola, cowpox and vaccinia viruses [3,4]. Mpox was once endemic to the African continent, but has now become a global health problem in more than 100 non-endemic countries [5]. According to a WHO report, from January 2022 to August 2024, more than 120 countries were affected by Mpox, with over 100,000 confirmed cases and more than 220 deaths [6]. Mpox is a double-stranded DNA virus comprising 197,000 genome base pairs, making it considerably larger than other viruses. There are two main clades: Clade I (Central African clade) and Clade II (West African clade). Among these, Clade I is the most virulent, resulting in frequent outbreaks and higher death rates. This strain is responsible for the resurgence of Mpox infections in India, China, Thailand, and Pakistan. It spreads rapidly and poses serious health risks, including skin rash or mucous membrane rash accompanied by fever, headache, muscle pain, back pain, lack of energy, and swollen lymph nodes [6]. The symptoms of infection can last for 2 to 4 weeks [6]. It spreads through the infected person, particularly through close contact with the skin, face, and mouth (Figure 1). In 2022, the Mpox outbreak primarily affected sexually active communities, causing rashes in unexpected and hard-to-notice areas such as anus and genitals, including the vagina, mouth, and throat [6,7]. Currently, there are no effective vaccines or drug candidates for controlling viruses and their pathogenic complications. As a result, this research aimed to identify effective drug candidates from the well-known polyherbal formulation, *Kabasura Kudineer*, which was recommended for SARS-CoV-2 during pandemic periods. The Ministry of the AYUSH (Govt. of India) recognises and promotes the traditional medical systems of medicine in India, including Ayurveda, yoga, naturopathy, Unani, Siddha, Sowa-Rigpa, and homoeopathy, collectively known as AYUSH. The AYUSH system, specifically Ayurveda, Yoga, and Siddha medicine, originated in India. Although Siddha is less well-known than Ayurveda, the Siddha system of medicine originated in the southern Indian state of Tamil Nadu and is now practiced by Tamil ethnic communities worldwide [8]. In 2015, the Siddha herbal formulation, Nilavembu Kudineer has garnered significant attention in Tamil Nadu for the treatment of dengue fever and chikungunya. Likewise, numerous formulations have been employed to enhance immune function and modulate inflammatory responses [8].

The National Advisory Board of India (AYUSH) recently recommended *Kabasura Kudineer*, a Siddha herbal formulation, for the management of asymptomatic and mild COVID-19. This formulation consists of 18 herbs including *Adhatoda vasica* Nees (leaves), *Anacyclus pyrethrum* (L.) Lag. (root), *Andrographis paniculata* (Burm.f.) Wall. ex Nees (whole plant), *Cissampelos pareira* L. (root), *Clerodendrum serratum* (L.) Moon (root), *Coleus amboinicus* Lour (leaves), *Cyperus rotundus* L. (root tuber), *Hygrophila auriculata* (Schumach.) Heine (root), *Ocimum sanctum* L. (leaf), *Piper longum* L. (fruit), *Plumbago zeylanica* L. (leaf), *Saussurea lappa* (Decne.) Sch.Bip (root), *Syzygium aromaticum* (L.) Merr. & L.M.Perry (flower), *Terminalia chebula* Retz (fruit rind), *Tinospora cordifolia* (Willd.) Hook.f. & Thomson (stem), *Tragia involucrata* L. (root), *Withania somnifera* (L.) Dunal (bark), and *Zingiber officinale* Roscoe (rhizome) (https://jammi.in/blog/kabasura-kudineer-benefits-ingredients-and-side-effects/) (accessed on 5 September 2024). During the coronavirus pandemic, this formulation has proven effective in reducing the complications associated with the virus, leading to its widespread use among individuals both pre- and post-infection.

Currently, the Mpox virus causes serious health complications worldwide. Hence, before it escalates into a severe outbreak, it is crucial to re-evaluate the antiviral effectiveness of this herbal formulation against the key proteins of the Mpox virus. As a novel drug is urgently needed for this virus, current research aims to determine the antiviral potential for Mpox in the recommended Siddha formulation using diverse drug screening approaches. In silico drug design is crucial for drug development and provides a rapid and efficient approach for the identification and development of small molecule drugs against viral infections. It enables the rapid and virtual screening of millions of compounds to identify potential drugs. Furthermore, it facilitates the prediction of metabolic pathways and potential toxicity of approved drug candidates [9]. This study used a variety of drug development techniques, including molecular docking, molecular dynamics simulations, binding free energy calculations, frontier molecular orbitals, and toxicity analysis, to evaluate the effectiveness of prescribed herbal formulations against the virus.

## 2. Materials and Methods

### 2.1. Phytochemicals and Proteins

**Figure 2:**
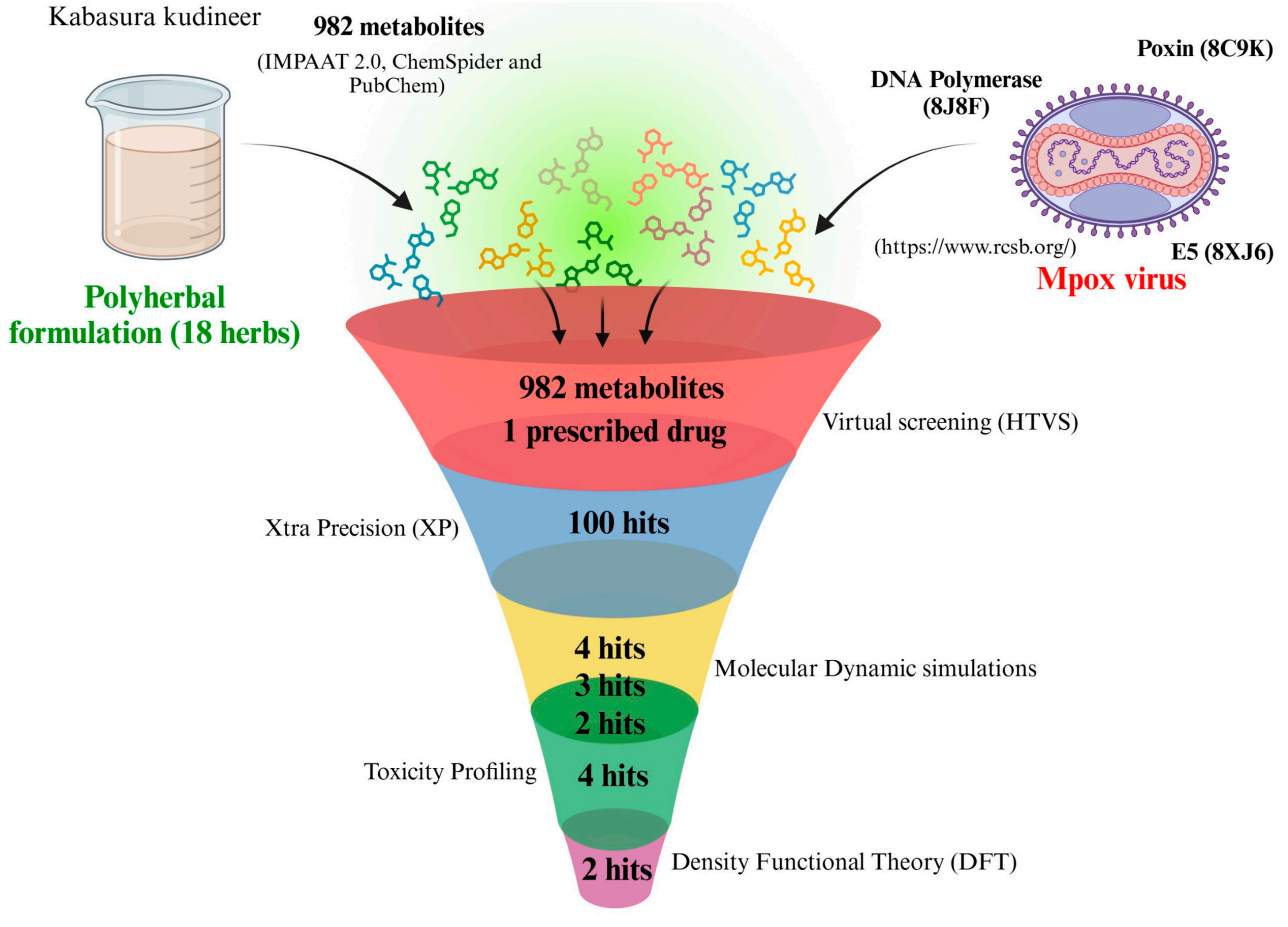
Schematic representation of workflow (Prescribed drug stands for Tecovirimat) (Created with www.BioRender.com).

The chemical components of the 18 herbs were sourced from IMPPAT 2.0 in accordance with the ingredients of *Kabasura Kudineer*. We retrieved nearly 982 chemical constituents as mole files for docking with Mpox proteins (Figure 2). The antiviral effects of *Kabasura Kudineer* on the Mpox virus were investigated by targeting Poxin, E5, and DNA polymerase. Poxin is essential for the survival and replication of the virus in host cells [10]. The Mpox virus E5, functioning as a helicase-primase, plays a vital role in DNA replication; nevertheless, the underlying molecular mechanisms are not yet fully understood [11]. The DNA polymerase holoenzyme of the mpox virus (MPXV) serves as an essential element in the genome replication. The crystallographic structures of these proteins were retrieved from the Protein Data Bank, with accession codes: 8C9K [10], 8XJ6 [12], and 8J8F [13].

### 2.2. Ligand Preparation

The collected molecules were prepared using the LiggPrep module (3.4). We achieved the original configuration of the gathered molecules by using the refined potential within the OPLS-2005 force field during the liquid simulation, which tackled the intricate physical characteristics of these molecules such as stereochemistry, ionisation, atomic composition, and chirality. All these processes were completed successfully, according to our previous investigation.

### 2.3. Protein Preparation and Site Analysis

During this stage, the protein preparation wizard module was used to examine the missing residues. The redundant chains, water molecules, and hetero atoms were eliminated. There are two gears, namely optimization and minimization, that were applied. The overlapping hydrogens were corrected using an optimization process, whereas the protein was stabilized using a minimization process after the water molecules were removed. The sitemap module was subsequently employed on the prepared proteins to identify the favorable site for ligand binding. The protein has multiple binding pockets, but not all of them are suitable for ligand binding. The module known as SiteMap was employed to identify the most active site for ligand docking. The druggable site was chosen based on the site score and volume derived from this evaluation.

### 2.4. Grid Generation and Molecular Docking

To stabilize the active site in the selected Mpox protein for binding the chemical components obtained from *Kabasura Kudineer*, grid boxes with sizes of X: 172.38, Y: 167.47 and Z: 198.01 for Poxin, X: 169.9, Y: 163.1 and Z: 146.83 for DNS polymerase and X: 12.92, Y: 7.14 and Z: −39.96 for E5 were constructed using glide grid module. To identify the active metabolites from *Kabasura Kudineer* that effectively reduce pathogenicity, replication, and virulence factors essential for survival in the host, molecular docking was conducted using the Grid-Based Ligand Docking module. The van der Waals radii of receptor atoms at 1.00 was used with a partial charge cutoff of 0.25 to reduce the potential in the target region. A grid at the centroid of the binding site was established and an extra precision docking protocol was used to position the prepared ligands within the binding site of the target protein. Glide generated the conformation, allowed all conformations within a series of filters, and selected the ultimate best-docked pose based on the scoring function parameters [14].

### 2.5. Molecular Dynamics (MD) Simulations

The binding stability of the compounds obtained from the polyherbal formulation with the Mpox protein was assessed for a simulation period of 100 nanoseconds. Molecular dynamics simulations were performed using the Desmond module V6.8 with the OPLS5 force field. It was used to investigate the binding stability of protein-ligand complexes, particularly those that demonstrated higher binding affinities with targets in molecular docking research. Initially, the system setup module was used to fix the orthorhombic water at buffer distances of a: 10.0 Å, b: 10.0 Å, and c: 10.0 Å and solvated with SPC water models. We then added 0.15 M Na^+^ Cl^−^ salt to neutralize the system. The particle mesh Ewald method was used to determine long-range electrostatic interactions; while the short-range van der Waals and Coulomb interactions were measured with a cutoff radius of 20.0 Å. The Nosè-Hoover chain thermostat and Martyna-Tobias-Klein barostat method were employed to maintain a temperature of 310 K and pressure of 1 atm. Thereafter, the simulated interaction tool was employed to generate plots for the RMSD, RMSF, residue contacts, contact summaries, radius of gyration, and other metrics [15].

### 2.6. Molecular Mechanics with Generalized Born and Surface Area Solvation (MM-GBSA) Calculations

We used the Prime Module to analyze the binding free energies for all the simulated complexes every 20 ns for a period of 100 ns.

∆G_bind_ = ∆E + ∆G_solv_ + ∆G_SA_ (1)

∆E = E_complex_ − E_protein_ − E_ligand_ (2)

where E_complex_, E_protein_ and E_ligand_ are the minimized energies of the protein = inhibitor complex, protein and inhibitor, respectively.

∆G_solv_ = G_solv_ (Complex) − I − G_solv_(protein) − G_solv_(ligand) (3)

where G_solv_ (Complex), G_solv_ (protein) and G_solv_ (ligand) are the solvation free energies of the complex and protein inhibitor, respectively.

∆G_SA_ = G_SA_ (complex) − G_SA_ (protein) − G_SA_ (ligand) (4)

where G_SA_ (complex), G_SA_ (protein) and G_SA_ (ligand) are the surface area energies of the complex, protein, and inhibitor, respectively [16].

### 2.7. Density Functional Theory (DFT)

Kenichi Fukui developed the frontier molecular orbital (FMO) theory, also known as the Fukui functions, in the 1950s. It reveals the highest energy occupied molecular orbital (HOMO) and lowest energy unoccupied molecular orbital (LUMO) of organic molecules. The FMO of a molecule represents the boundary of an electron, which plays a crucial role in defining the energy gap between the HOMO and LUMO [17]. The highest occupied molecular orbital (HOMO) primarily acts as an electron donor, while the lowest unoccupied molecular orbital (LUMO) serves as an electron acceptor. The interplay between the electron donor and acceptor pair can dominate other chemical reactivities of a molecule. During the electrophilic-nucleophilic reaction, electrons move from the HOMO to the LUMO. This makes the two molecular orbitals have different energies. This gap highlights the photochemical behaviour of transition metal complexes within organic molecules as well as their resilience and stability. To understand the atomic reactivity of hit molecules with respect to electrophilic and nucleophilic interactions, the energies of the HOMO and LUMO were assessed using Schrödinger Jaguar V13.2. The energy difference between the two molecular orbitals (HOMO-LUMO gaps) was calculated using the following formula:

ΔE = E_LUMO_ − E_HOMO_

where E is the HOMO-LUMO gaps, E_LUMO_ is the lowest unoccupied molecular orbital energy and E_HOMO_ is the highest occupied molecular orbital energy.

### 2.8. Toxicity Assessment

An internet-based toxicity tool, ProTox-3.0, was used to investigate the toxicity profiles (organ toxicity and toxicity endpoints) of the compounds exhibiting higher binding affinities with the Mpox viral proteins. The SMILES of the hit compounds were extracted from the docked file to examine these profiles. Subsequently, we separately applied the collected SMILES to the portal to determine the toxicity profiles of these molecules [18].

## 3. Results and Discussion

### 3.1. Effects of Herbs in Antiviral Effects

In recent decades, scientific research has identified several synthetic antiviral drugs that are effective against various viruses. However, some of these drugs such as arbidol [19], favipiravir [20], ledipasvir [21] and sialic acid derivatives [22] cause side effects and in certain cases, lose their effectiveness against evolving viral resistance strains [23]. Medicinal plants and their chemical substances are valuable resources for the development of novel antiviral drugs [24]. Traditional herbal therapy based on indigenous practices has a long history in the treatment of many chronic and infectious diseases [25]. Consistent with the above claims, two polyherbal formulations, Nilavembu Kudineer and *Kabasura Kudineer* have been used in Ayurvedic medicinal practices to reduce the health effects of dengue fever, chickenpox, and coronaviruses. Therefore, recent scientific studies have searched for antiviral compounds from various medicinal plants that are used in traditional herbal practices. The present research also re-evaluates the antiviral potential for the monkeypox virus from the chemical components of *Kabasura Kudineer*. In order to screen the most active chemical components of *Kabasura Kudineer* against E5, poxin and DNA polymerase, a high-throughput virtual screening approach was used. It was found that most of the chemical components of this formulation had good binding affinities with the target molecules, which represents the drug effectiveness of these chemical constituents for the complications associated with E5, poxin and DNA polymerase. The top 100 molecules of each protein were further examined to more precisely identify their contacts using the Xtra Precision docking module. The present research found that the most effective chemicals in this polyherbal formulation were chlorogenic acid, chebulic acid, rosmarinic acid, and citric acid for E5, while caffeic acid, citric acid, and plumbagic acid for poxin and plumbagic acid and delphinidin for DNA polymerase.

### 3.2. Binding Affinities of Herb Compounds for E5

#### 3.2.1. Chlorogenic Acid

**Figure 3:**
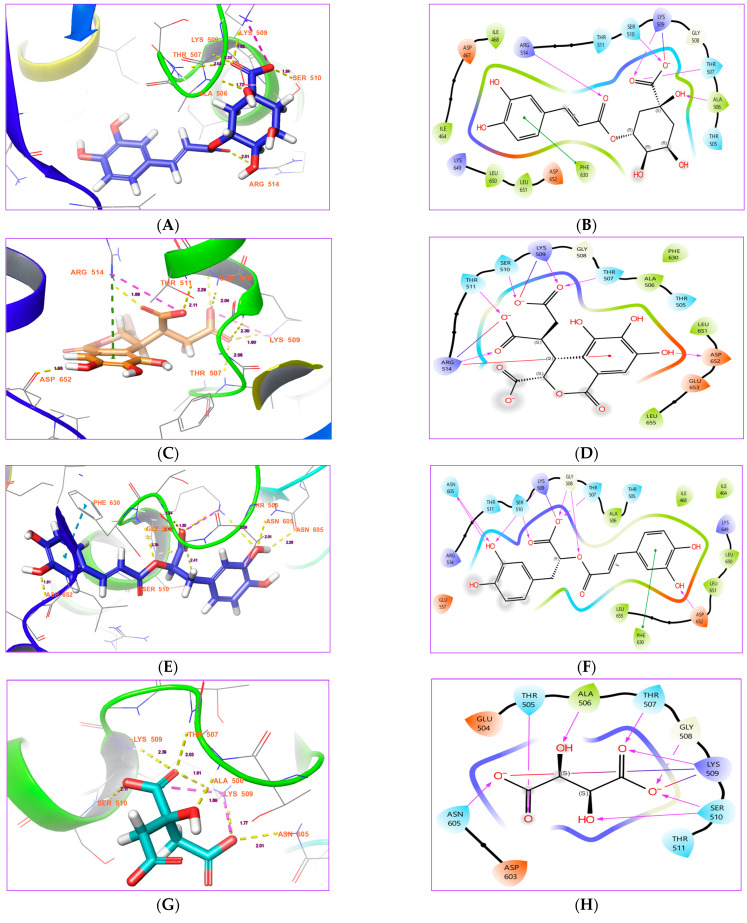
Binding affinities of organic acids from polyherbal formulation with Mpox E5: (**A**,**B**): Chlorogenic acid, (**C**,**D**): Chebulic acid, (**E**,**F**): Rosmarinic acid and (**G**,**H**): Citric acid.

Chlorogenic acid has stronger binding affinity with a docking score of −13.3289 kcal/mol (Table 1). It consists of seven interactions with monkeypox virus E5, comprising 5 hydrogen bonds, 1 salt bridge, and 1 pi-pi stacking interaction (Figure 3A,B). The key residues of the binding site that have interactions with chlorogenic acid are ARG 514, SER 510, LYS 509, THR 507, ALA 506, and PHE 630 (Table 2). Of these, PHE 630 has a pi-pi stacking contact with chlorogenic acid while LYS 509 has salt bridge contact (Figure 3A). The distances of the hydrogen bond contacts are 2.01 Å (ARG 514), 1.84 Å (SER 510), 2.33 Å (LYS 509), 2.53 Å (THR 507), and 1.72 Å (ALA 506), respectively. Except for ALA 506, all residues formed hydrogen bonds with the oxygen moiety of chlorogenic acid (Figure 3B). Previously, the strong antiviral properties of this molecule were shown in both experimental and computational studies. Ding et al. [26] reported that it effectively reduces the cell growth of influenza A (H1N1/H3N2) virus, with an EC50 value of 44.87 μM. It also inhibits the release of new viral particles by reducing the growth of nucleoprotein. In an in vivo experiment, the treatment with chlorogenic acid successfully reduced DNA breakage in hepatitis B virus [27]. It also reduced the population of viruses in HBV-induced animals. Chiang et al. [28] stated that it was effective against many viruses, including HSV-1, HSV-2, ADV-3, and ADV-11, with EC50 values of 15.3 μg/mL, 87.3 μg/mL, 14.2 μg/mL, and 13.3 μg/mL. It was found to possess anti-enterovirus properties, inhibiting viral replication, transcription, and translation. At a concentration of 6.3 µg/mL, it successfully suppressed HBV replication [29]. It also modulates the expression of various signaling pathways linked to host survival and replication, including IL-6, TNF-α, IFN-γ, and MCP-1 [29].

#### 3.2.2. Chebulic Acid

Chebulic acid had a docking score of −11.3933 kcal/mol (Table 1). It consists of nine interactions with the monkeypox virus E5, comprising 6 hydrogen bonds, 2 salt bridges, and 1 pi-cation interaction (Figure 3C,D). Assessment of the docked complex showed that THR 511, SER 510, LYS 509, LYS 507, ARG 524, and ASP 652 are the residues that interact with chebulic acid (Table 2). All of these residues, with distances of 2.11 Å, 2.04 Å, 1.90 Å, 2.06 Å, 1.89 Å, and 1.98 Å, exhibited hydrogen bond interactions with chebulic acid (Figure 3C). Even though these residues have hydrogen bond interactions, some of them, notably LYS 509 and ARG 514, also have additional interactions, such as salt bridges and pi-cation interactions. All of these residues, in contrast to the chlorogenic acid-Mpox E5 complex, have established hydrogen bond interactions with the oxygen moiety of chebulic acid alone (Figure 3D). Previous studies have documented its effectiveness against multiple viruses, such as SARS-CoV-2 [30], COVID-19 [31], HIV [32], Dengue virus [33], and Chikungunya virus [34]. In-silico findings of Vora et al. [34] suggested that chebulic acid might act as an inhibitor of the envelope glycoprotein GP120, reverse transcriptase, and protease of HIV because of its strong binding affinity and good stability with the key protein of HIV. According to Khan et al. [35], it has good binding affinities with the SARS-CoV-2 spike glycoprotein and docking scores of −8.5 kcal/mol. This provides further evidence that chebulic acid possesses antiviral properties. It has been identified to be efficient against the dengue virus because of its significant binding affinity for NS5 methyltransferase [33].

#### 3.2.3. Rosmarinic Acid

Rosmarinic acid has a docking score of −9.8999 kcal/mol (Table 1). It consists of nine interactions with the monkeypox virus E5, comprising eight hydrogen bonds, one salt bridge, and one pi-pi stacking interaction (Figure 3D,E). The analysis of rosmarinic acid in the binding site of Mpox E5 revealed that the residues ASN 605, LYS 509, LYS 410, GLY 508, ASP 652, THR 507 and PHE 630 interacted with rosmarinic acid (Table 2). Of these, residues such as ASN 605, LYS 509 and GLY 508 had two interactions each (Figure 3D). Even though these residues have hydrogen bond interactions, some of them, notably LYS 509 and PHE 630, also have additional interactions, such as salt bridges and pi-pi stacking interactions. The distances of the hydrogen contacts were measured to be 2.26 Å, 2.01 Å, 2.59 Å, 2.41 Å, 2.35 Å, 1.08 Å, 1.54 Å and 1.91 Å (Figure 3D), respectively. The oxygen and hydrogen groups of rosmarinic acid have double bond interactions with residues ASN 605, LYS 509, and GLY 508 (Figure 3E). Numerous studies have indicated that rosmarinic acid is a potential agent against various viruses, including Dengue [36,37,38], Influenza A H1N1 [39], hepatitis B [40], enterovirus 71 [41], Rift Valley fever [42], HIV-1 [43], herpes [37] and Nipah virus [44]. A recent in silico study reported that it can also inhibit the Mpox virus by targeting the E8L protein of the monkeypox virus (MPXV) [45]. The binding affinity score of this molecule for the E8L protein of Mpox was −156.51 kcal/mol. In 2020, cell-based antiviral research and in vivo mouse model studies documented that rosmarinic acid effectively inhibited the replication of Enterovirus A71. Hsieh et al. [46] reported that RA may have an effect on the early stages of a viral infection by interacting directly with viral particles and breaking down the interactions between the virus and PSGL1 and between the virus and heparan sulfate, while leaving the interactions between the virus and SCARB2 alone. They indicated that it is a multi-target drug that targets EV-A71, PSGL 1, and heparan sulfate [46].

#### 3.2.4. Citric Acid

Citric acid has a docking score of −9.59471 kcal/mol (Table 1). It has nine hydrogen bonds and two salt bridge contacts with the Mpox E5. In this docked complex, the residues THR 505, ALA 506, THR 507, ASN 605, LYS 509, and SER 510 formed strong hydrogen bonds with citric acid (Table 2 and Figure 3G). The distances of the hydrogen contacts were measured to be 2.94 Å, 1.09 Å, 2.03 Å, 2.01 Å, 1.77 Å, 2.02 Å, and 2.11 Å, respectively. Although all these residues have hydrogen bond interactions, LYS 509 has three interactions with citric acid, comprising two hydrogen bonds and one salt bridge. All of these residues established hydrogen bond interactions with the oxygen and hydroxyl moieties of citric acid (Figure 3H). Recent research reported that citric acid suppressed the proliferation of COVID-19 cells with an IC50 value of 16.27 ± 0.02 µg/mL [47]. In silico research has shown high binding affinities for non-structural protein 12 (7AAP) and envelope small membrane protein (7K3G) of SARS-CoV-2, implying that it may act as an inhibitor of these protein-related complications [47]. In silico studies have indicated that citric acid can impede the dengue virus, chikungunya virus, HIV-1, and influenza virus by disrupting the functions of their essential proteins, including S2B/NS3 protease and methyltransferase (*Dengue virus*), nsP2 protease (*Chikungunya virus*), protease and reverse transcriptase (HIV-1), and neuraminidase (*Influenza virus*) [48].

#### 3.2.5. Tecovirimat

**Figure 4:**
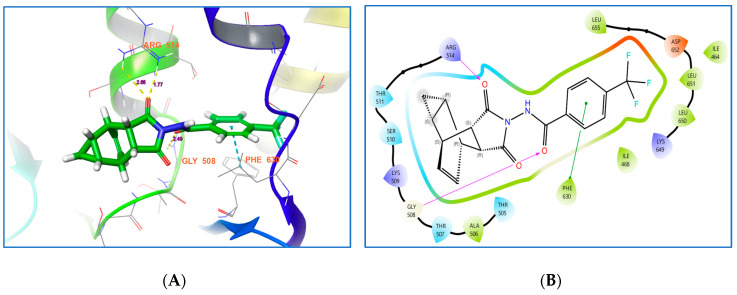
(**A**,**B**) Binding affinities of tecovirimat with Mpox E5.

The prescribed antiviral drug, Tecovirimat, has three contacts with the residues of E5. It features two hydrogen bond contacts and a Pi-Pi stacking contact with ARG 514, GLY 508 and PHE 630, respectively (Table 2 and Figure 4A,B). This molecule has minimal binding affinities compared to chlorogenic acid, chebulinic acid, rosmarinic acid and citric acid, suggesting that it may show a stronger antiviral effect on Mpox than the prescribed drug in in vitro or in vivo studies.

### 3.3. Stability of Hit Molecules-E5

#### 3.3.1. Chlorogenic Acid-Mpox E5

**Figure 5:**
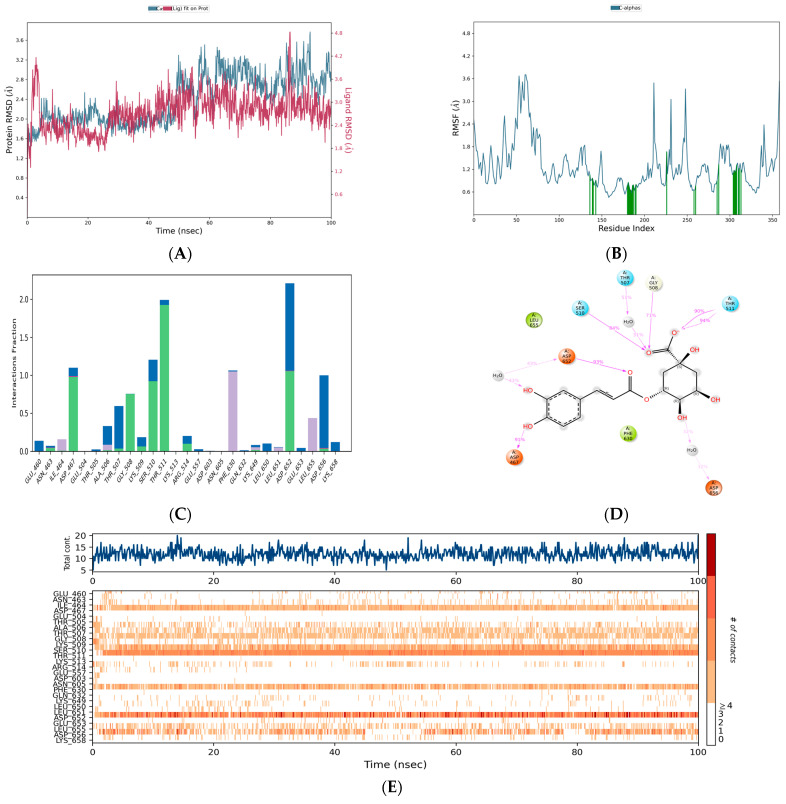
Simulation plots represent the stability of Chlorogenic acid-Mpox E5 complex: (**A**) RMSD, (**B**) RMSF, (**C**) Interaction fraction plot, (**D**) Contact summary and (**E**) Chlorogenic acid contacts plot with the residues of Mpox E5.

The c-α of Mpox E5 was stable with chlorogenic acid. Notably, c-α of the target protein overlapped within the RMSD range of chlorogenic acid (Figure 5A). Stability was maintained within an RMSD range of less than 2.0 from 0 ns to 47 ns. Subsequently, a deviation occurred at 48 ns, reaching an RMSD range of 3.2 Å. The plot shows irregular RMSD peaks, with a maximum range of 3.2 Å and a minimum of 2.4 Å. However, the c-α of the target was found to be stable with an RMSD range of 2.8, notably at the periods of 90 ns–100 ns. During the simulated periods, the residues of Mpox E5, including CYS 385, LEU 384, LYS 377, PRO 386, GLY 703, SER 381, ARG 387, PRO 542, GLU 378, ASN 593, GLU 382, PRO 376, SER 566, SER 380, and LEU 383, have exhibited significant fluctuations reaching up to 3.703 Å, 3.70 Å, 3.67 Å, 3.57 Å, 3.52 Å, 3.51 Å, 3.51 Å, 3.48 Å, 3.35 Å, 3.32 Å, 3.14 Å, 3.12 Å, 3.05 Å, 3.02 Å and 3.02 Å (Figure 5B). Of them, ASP 652 has ionic and water-bridge assisted hydrogen bond contact with the fraction range of 2.1 Å followed by THR 511 (2.0 Å: water assisted hydrogen bond contacts) and SER 510 (1.02 Å) and ASP 167 (1.01 Å) (Figure 5C), respectively. Chlorogenic acid exhibited significant interactions with Mpox E5 residues, with binding percentages of 94% and 90% for THR 511, 93% and 43% for ASP 652, 91% for ASP 467, 84% for SER 510, 71% for GLY 508, and 32% for ASP 656 (Figure 5D,E), respectively.

#### 3.3.2. Chebulic Acid-Mpox E5

**Figure 6:**
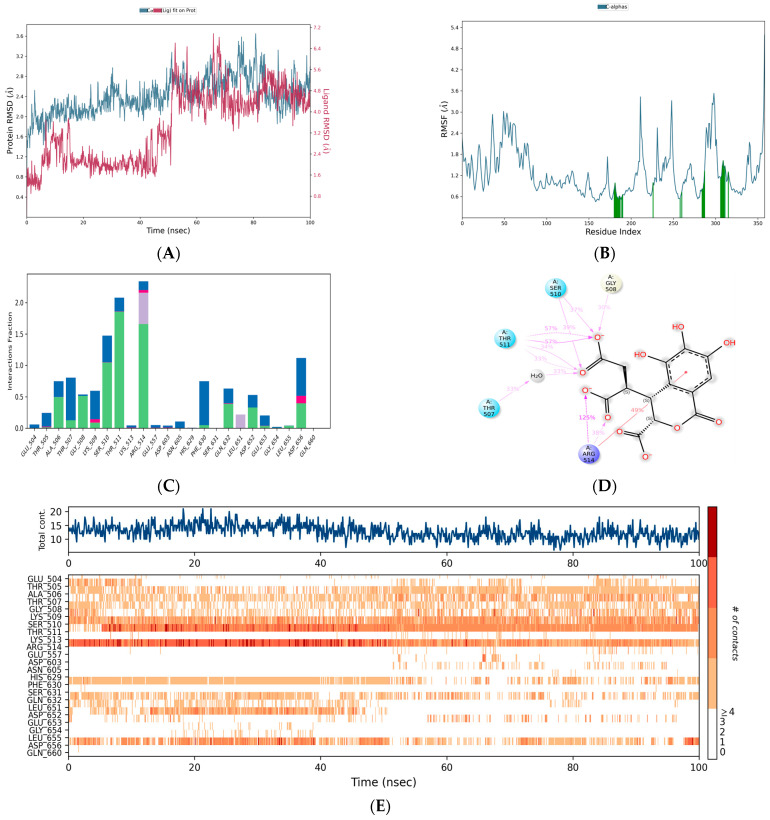
Simulation plots represent the stability of Chebulic acid-Mpox E5 complex: (**A**) RMSD, (**B**) RMSF, (**C**) Interaction fraction plot, (**D**) Contact summary and (**E**) Chebulic acid contacts plot with the residues of Mpox E5.

Chebulic acid also showed good compatibility with Mpox E5, similar to chlorogenic acid. The initial RMSD for this compound was 1.6 Å, which subsequently increased, achieving an RMSD of 2.4 Å at 50 ns (Figure 6A). At 52 ns, a sudden deviation appeared, reaching an RMSD range of 2.5 Å. It then reached an RMSD range of 3.2 Å at 77 ns. However, it switched to an RMSD of 2.4 at 84 ns and maintained this state until 100 ns with a ligand-overlapping RMSD, indicating a strong interaction between chebulic acid and Mpox E5. The RMSF graph showed that GLY 703, ASP 643, PRO 542, ASN 593, ASN 641, ASN 642, ALA 644, ILE 702, and HIS 373 were the most fluctuating areas, with fluctuation ranges of 5.181, 3.531, 3.43, 3.323, 3.252, 3.208, 3.177, 3.096, and 3.018 Å, respectively (Figure 6B). Among these, the residues, ARG 514, ASP 656, THR 511, SER 510, GLN 632, GLU 633, ASP 603, and LYS 513, have established hydrogen bond contacts with chebulic acid with the assistance of water bridges and ions (Figure 6C). Notably, ARG 514 has the highest interaction fraction range of 2.03 Å followed by THR 511 (2.01 Å) and SER 510, respectively. The contact summary and contact plots of chebulic acid and Mpox E5 revealed the degree of their interaction across the simulated periods (Figure 6D,E).

#### 3.3.3. Rosmarinic Acid-Mpox E5

**Figure 7:**
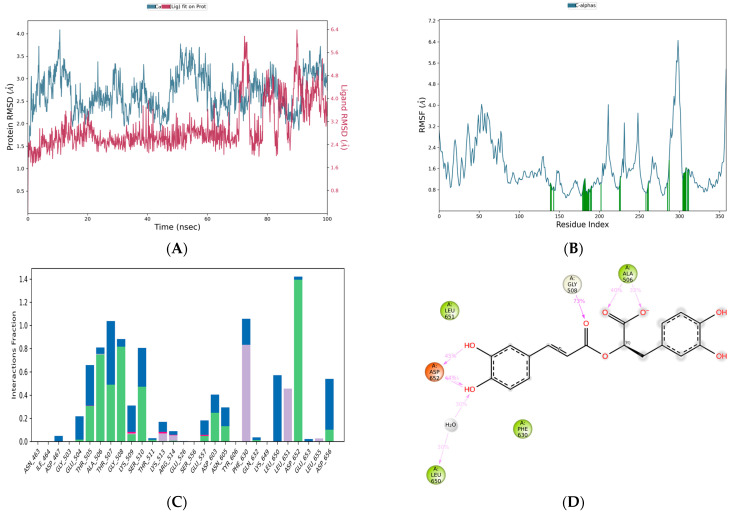

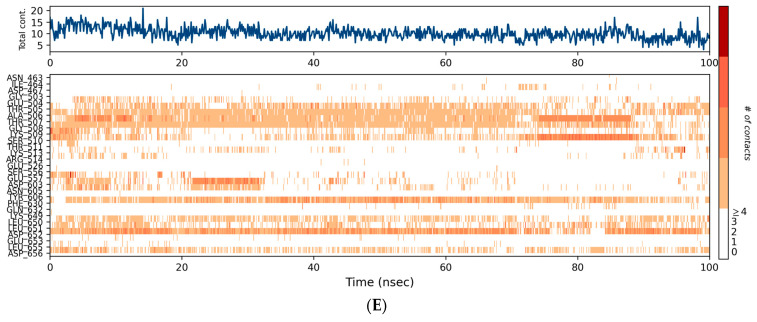
Simulation plots represent the stability of Rosmarinic acid-Mpox E5 complex: (**A**) RMSD, (**B**) RMSF, (**C**) Interaction fraction plot, (**D**) Contact summary and (**E**) Rosmarinic acid contacts plot with the residues of Mpox E5.

In this case, the RMSD range of the protein varied significantly with the chlorogenic acid and chebulic acid complexes. Initially, it achieved an RMSD range of 3.5 Å, with a gradual divergence from 0 to 16 ns. At 17 ns, it turned out to be stable, showing an RMSD of <2.8 Å (Figure 7A). This deviation remained consistent for 45 ns, with a small deviation range of 3.2 ns from 25 ns to 32 ns. There was a higher deviation from 48 to 60 ns, reaching an RMSD range of 3.7 Å. The overlapping RMSD of the ligand and protein from 60 ns to 100 ns in the simulated timeframes shows that rosmarinic acid strongly binds with Mpox E5. Residues ASP 643, ALA 644, ASN 641, ASN 642, GLY 703, GLU 640, ALA 638, TYR 645, and ILE 702 were identified as impacted residues, which fluctuated in the ranges of 6.455, 5.707, 5.684, 5.633, 3.967, 3.75, 3.724, 3.703, and 3.687 Å, respectively (Figure 7B). In this case, the residues with elevated interaction fractions possess water bridge-assisted hydrogen bond interactions. Notably, ASP 652, THR 507, GLY 508, and ALA 506 had interaction fraction ranges of 1.4 Å, 1.05 Å, 0.9 Å, and 0.8 Å, respectively. Some of the residues, such as LYS 509, LYS 513, GLU 557, and ARG 514, established water bridge, hydrophobic, and ionic-assisted contacts (Figure 7C). Rosmarinic acid has a strong degree of contact with GLY 508 (73%), ALA 506 (40% and 33%), ASP 652 (45% and 44%), and LEU 650 (30%), according to the contact summary and contact plots (Figure 7D,E), which shows that the rosmarinic acid-Mpox E5 complex is stable.

#### 3.3.4. Citric Acid-Mpox E5

**Figure 8:**
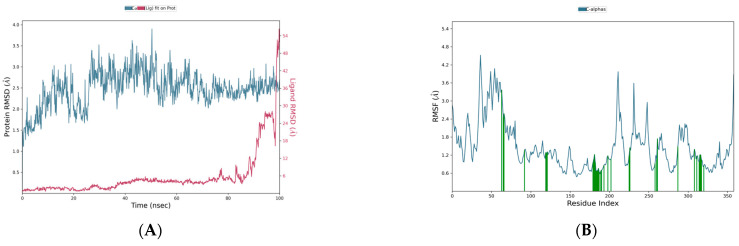

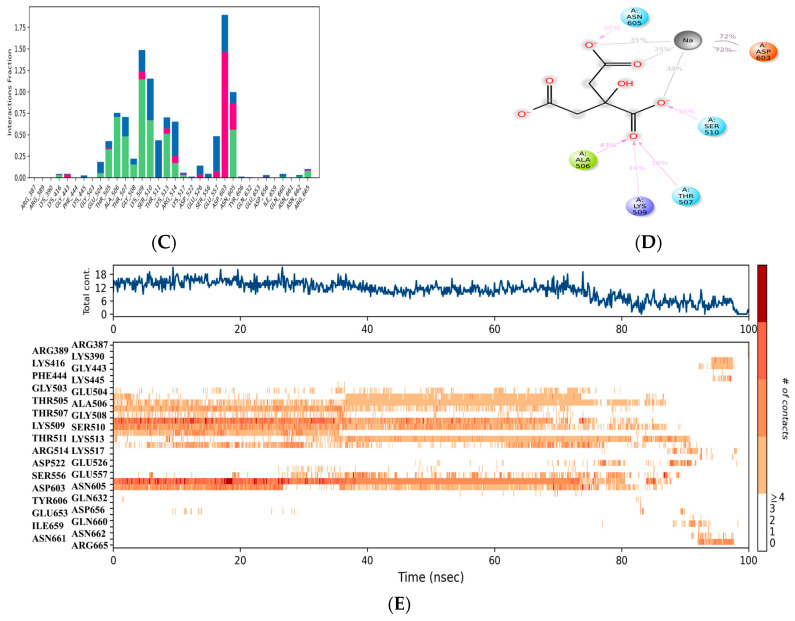
Simulation plots represent the stability of Citric acid-Mpox E5 complex: (**A**) RMSD, (**B**) RMSF, (**C**) Interaction fraction plot, (**D**) Contact summary and (**E**) Citric acid contacts plot with the residues of Mpox E5.

The simulation results of citric acid and Mpox E5 complex revealed that C-α was stable with an average RMSD range of 2.6 Å. From 0 ns to 24 ns, the RMSD remained around 2.5 Å, then at 26 ns, it increased to 3.0 Å and remained stable until 64 ns (Figure 8A). The RMSD plot indicated that c-α was highly compatible from 65 to 100 ns, with an RMSD range of 2.5 Å. The RMSF data revealed that the residues GLU 360, GLU 378, HIS 373, PRO 542, GLY 703, and GLU 361 had notable alterations, with the ranges of 4.519 Å, 4.074 Å, 3.979 Å, 3.97 Å, 3.874 Å, and 3.87 Å (Figure 8B), respectively. The interaction fraction plot showed that LYS 509, ASN 605, SER 510, LYS 513, ARG 514, THR 505, and LYS 717 made contact with citric acid through water bridges and ionic-assisted hydrogen bonds (Figure 8C). Interestingly, the sodium ion (Na^+^) of Mpox E5 coordinates the binding affinities with citric acid, particularly the contacts between ASP 603 and the oxygen atoms of citric acid. It was found that there were two contacts with citric acid, each with a strength of 72%. The other residues ALA 506, THR 507, SER 510, LYS 509, and ASN 605 had a binding strengths of 43%, 36%, 35%, 34%, and 30% (Figure 8D,E), respectively.

#### 3.3.5. Tecovirimat-Mpox E5

**Figure 9:**
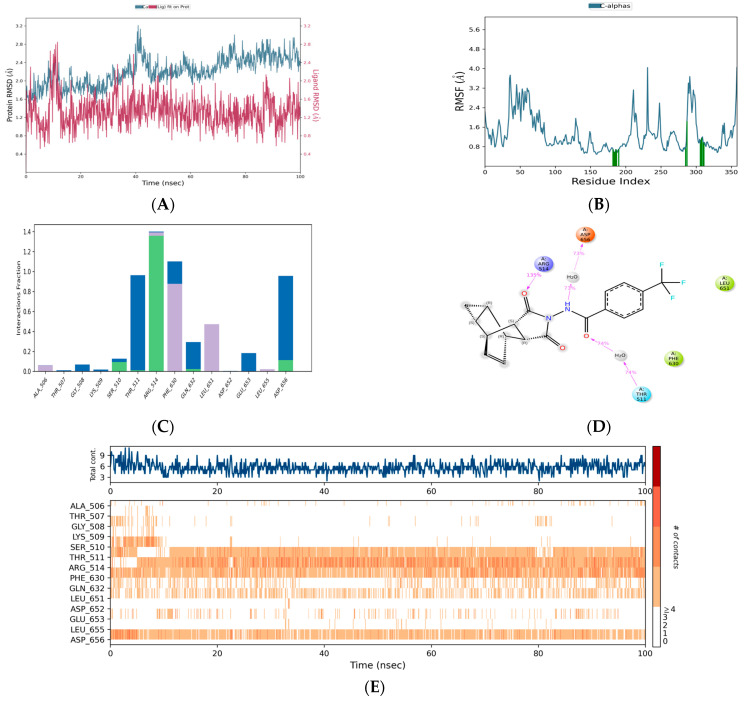
Simulation plots represent the stability of Tecovirimat-Mpox E5 complex: (**A**) RMSD, (**B**) RMSF, (**C**) Interaction fraction plot, (**D**) Contact summary and (**E**) Tecovirimat contacts plot with the residues of Mpox E5.

The simulation results of this complex revealed that the C-α of target had a stable RMSD range of <2.8 Å (Figure 9A). From 0 ns to 30 ns, an RMSD range of 2.0 Å. Subsequently, the deviation has progressively increased and suddenly attained an RMSD range of 2.8 Å at 40 ns. Later, RMSD range was 2.3 until 70 ns. However, it reached the value of 2.8 Å at 100 ns. During the simulation periods, the residues GLY 703, SER 566, GLU 360, ARG 636, SER 359, SER 634, LEU 369, LEU 384, LYS 377, GLU 637, PRO 542, ASN 641, PRO 386, GLU 361, CYS 385, SER 381, HIS 373, and GLU 378 exhibited significant mobility, with fluctuation ranges of 4.05, 4.042, 3.733, 3.675, 3.603, 3.41, 3.344, 3.199, 3.169, 3.148, 3.126, 3.124, 3.108, 3.102, 3.071, 3.067, 3.015, and 3.01 Å (Figure 9B), respectively. The interaction fraction plot revealed that ARG 515 had hydrophobic and water bridge assisted hydrogen bond contacts with tecovirimat showing the fraction range of 1.4 Å (Figure 9C). The contact summary and contact plots revealed that tecovirimat exhibited a significant contact strength, comparable to that of rosmarinic acid and citric acid (Figure 9D,E).

### 3.4. Per-Residue Energy Decomposition Analysis

To identify the residues critical for stabilizing the binding affinities with the mpox E5 protein, a per-residue energy decomposition analysis was conducted on the post-MD simulation complexes, specifically: chlorogenic acid-Mpox E5, chebulic acid-Mpox E5, rosmarinic acid-Mpox E5, and citric acid-Mpox E5 (Table 3).

### 3.5. Radius of Gyration (Rg)

**Figure 10:**
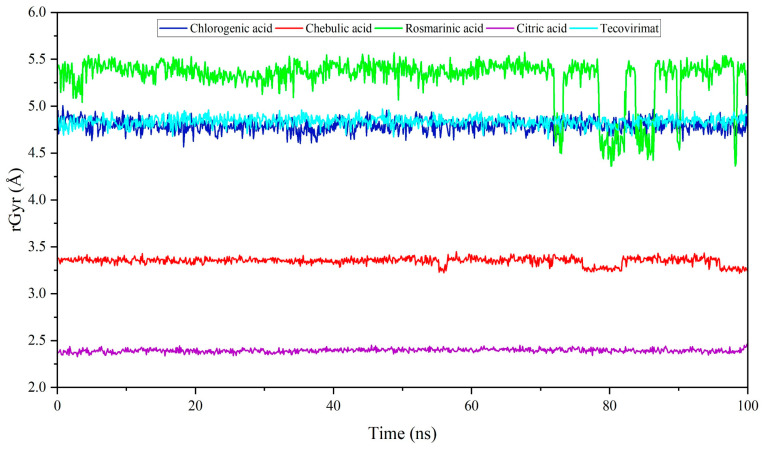
Plot represents the compactness of organic acids and a prescribed drug tecovirimat.

The compactness of chlorogenic acid, chebulic acid, rosmarinic acid, citric acid, and tecovirimat was assessed with Mpox E5 using the radius of gyration (Figure 10). This measurement quantifies the distance from the axis of the structure to the atomic site where energy transfer occurs during rotation [49]. This study found that chlorogenic acids and tecovirimat have identical Rg value (4.83 Å) throughout the simulation periods of 100 ns. Chebulic acid, another leading hit metabolite against E5, had an average Rg value of 3.7 Å. On the other hand, the rosmarinic acid has an average Rg value of 5.3 Å. However, a deviation occurred from 73 to 100 ns, resulting in intermittent Rg values of 5.0 Å. Citric acid had an average Rg value of 2.4 Å throughout the simulation period, which is much lower than that of other acids.

### 3.6. Efficacy of Polyherbal Compounds in Mpox Poxin-Associated Complications

The acids in this herbal formulation, particularly caffeic acid, citric acid, and plumbagic acid, demonstrated docking scores of −8.49023, −6.80386, and −5.91719 kcal/mol (Table 4), respectively. Despite having the highest docking scores for this target among the docked metabolites, subsequent interaction analysis showed that these acids had fewer interactions with the target. In recent research, the antiviral activity of caffeic acid on the Ilhéus virus in Vero and A549 cell lines was investigated by Saivish et al. [50]. Based on the findings of in vitro studies, they concluded that caffeic acid has the potential to inhibit viral cell replication. It also exhibited a strong interaction with two viral proteins, mitogen-activated protein kinase 1 (4N0S) and human carbonic anhydrase (6YRI). It has also been reported to exert antiviral effects against herpes simplex virus, Ebola pseudotyped virus, and vaccinia virus. The EC50 values for these viruses were 27.2 ± 1.9 μM, 67.0 ± 3.3 μM, and 117 ± 3 μM, respectively [51]. Previously, Wang et al. [52] investigated the inhibitory effects of caffeic acid on hepatitis B (HBV) virus using both in vitro and in vivo approaches. This research pointed out that the reduction in DNA levels and concentrations of surface and e-antigens impacted by caffeic acid led to a 17.23% decrease in viral activity. It could also be effective against influenza A virus and poliovirus. For instance, Utsunomiya et al. [53] demonstrated that caffeic acid substantially inhibited the replication of influenza A virus.

**Figure 11:**
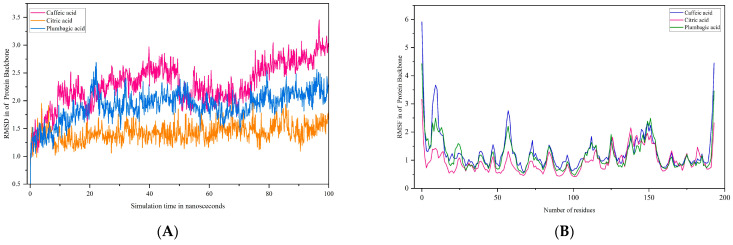
(**A**,**B**) Plots represents the RMSD and RMSF ranges of hit molecules with poxin.

In order to evaluate the stability of these acids at the binding site of poxin, molecular dynamics simulations were performed for 100 ns. The C-α atoms of poxin were stable up to 73 ns and had an RMSD range of less than 2.8 Å. Interestingly, a lower RMSD range of <2.5 Å was observed from 50 ns to 73 ns (Figure 11A). However, at 75 ns, a gradual deviation occurred, reaching an RMSD range of 3.0 Å when the simulation period reached 100 ns. With an RMSD of <1.5, the citric acid-poxin complex was extremely stable (Figure 11A). In this simulation complex, there was no significant deviation in the C-α of poxin from 0 ns to 100 ns. Another complex, namely plumbagic acid and poxin, had the lowest RMSD value, with an average of 1.8 Å (Figure 11A). This shows that plumbagic acid has excellent stability with the protein, which could be due to its strong binding affinity at the binding pocket.

**Figure 12:**
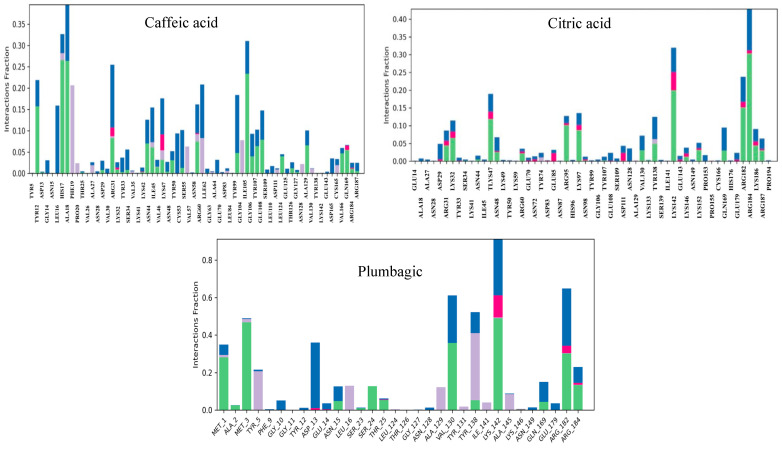
The plots depict the residues of poxin, which established ion, water-bridge, and hydrophobic-assisted hydrogen bond contacts with the chemical constituents of *Kabasura Kudineer* molecules during the simulation period.

The RMSF plot of the complex containing caffeic acid and poxin revealed that the most fluctuating residues of the target throughout the simulation period were MET 1, PRO 194, GLY 10, ALA 2, GLY 11, and PHE 9, with corresponding values of 5.912, 4.455, 3.669, 3.596, 3.528, and 3.345 Å (Figure 11B), respectively. On the other hand, we identified MET 1, PRO 194, and ALA 2 as highly motile residues in the plumbagic acid complex, exhibiting fluctuations of 4.432, 3.458, and 2.756 Å (Figure 11B), respectively. Nonetheless, MET 1 and ALA 2 maintained their connections with the target despite fluctuations caused by the simulation environment. The most motile residue in the citric complex was MET 1, which had an RMSF range of 3.168 Å (Figure 11B). The residue interaction fraction plots showed that poxin residues formed hydrogen bond contacts with caffeic acid, citric acid, and plumbagic acid, which were hydrophobic, water-assisted, and ion-assisted (Figure 12).

### 3.7. Efficacy of Polyherbal Compounds in Mpox DNA Polymerase-Associated Complications

Among the docked chemical components, plumbagic acid and delphinidin were found to be the most effective in inhibiting DNA polymerase-associated complications, with docking scores of −7.57867 and −7.55301 Kcal/mol (Table 5), respectively. Currently, there are no reports on the antiviral effect of plumbagic acid. However, delphinidin has been previously reported to have antiviral activity against various viruses, as discussed in this research. Previously, Vázquez-Calvo et al. [54] investigated the antiviral efficacy of delphinidin against West Nile, Zika, and dengue viruses. Their findings indicated that delphinidin significantly diminished viral infectivity and replication by interfering with the life cycle of these viruses. It has also been reported to be effective in reducing the replication and transmission of the Hepatitis C virus [55]. Recent research has shown that delphinidin-3-glucoside, a derivative of delphinidin, is effective against SARS-CoV-2, particularly in inhibiting its replication with an IC50 value of 35.8 ± 1.38 µM [56]. Akinnusi et al. [57] found that it binds strongly to several SARS-CoV-2 proteins, including 3CL protease (7JT7), RNA-dependent RNA polymerase (6M71), spike protein (6LZG), helicase (7NNG), and human ACE-2 (6LZG). This suggests that this molecule may be able to combat coronaviruses.

**Figure 13:**
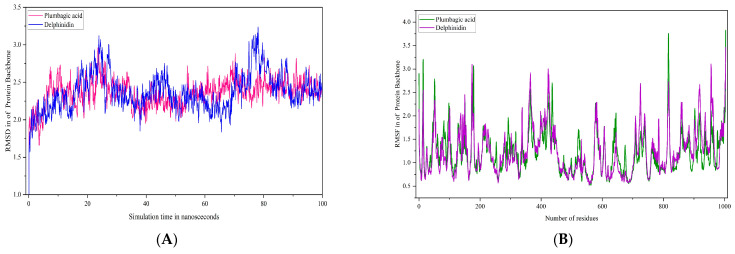
(**A**,**B**) Plots represent the RMSD and RMSF ranges of hit molecules with Mpox virus DNA polymerase.

**Figure 14:**
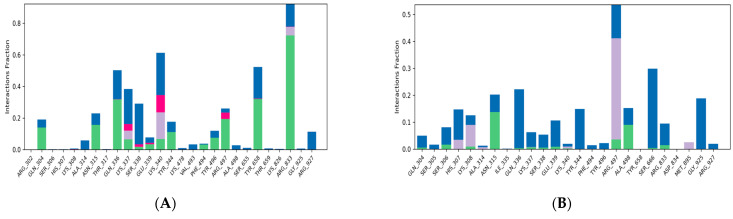
(**A**,**B**) The plots depict the residues of the DNA polymerase of Mpox virus, which established ion, water-bridge, and hydrophobic-assisted hydrogen bond contacts with the chemical constituents of *Kabasura Kudineer* molecules during the simulation period.

Molecular dynamics simulations indicated that the plumbagic acid and DNA polymerase complex were stable, with an RMSD of 2.5 Å (Figure 13A). The RMSD plot revealed that the complex containing delphinidin and DNA polymerase was also stable. In this case, the C-α of DNA polymerase has deviated up to an RMSD range of 3.0 and 3.2 Å at simulation periods of 23 ns and 77 ns, respectively. Notwithstanding these sudden fluctuations, they subsequently showed a stable range of <2.8 Å, signifying that delphinidin has noteworthy stability in the binding pocket (Figure 13A). The simulation results of plumbagic acid and DNA polymerase complex showed that the target residues, specifically TYR 1004, SER 817, ASN 816, GLU 14, PHE 179, and LYS 818, showed significant motion, reaching up to 3.827, 3.757, 3.341, 3.201, 3.069, and 3.018 Å (Figure 13B and Figure 14A), respectively. The simulation results of the delphinidin and DNA polymerase complex showed that TYR 1004, ARG 956, LYS 174, THR 423, and SER 957 were highly mobilized residues in the target during the simulation period, fluctuating up to 3.458, 3.104, 3.097, 3.004, and 2.997 Å, respectively (Figure 13B and Figure 14B).

### 3.8. HOMO and LUMO

**Figure 15:**
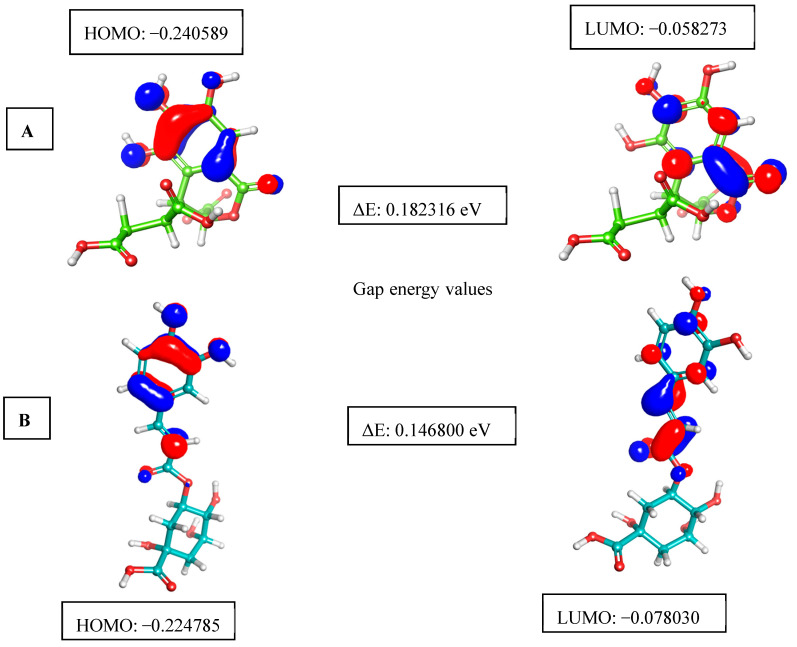
The picture depicts the asymmetric HOMO, LUMO, and HOMO-LUMO gap energies for two selected *Kabasura Kudineer* compounds ((**A**). Chebulic acid and (**B**). Chlorogenic acid)), with red representing negative phases and blue representing positive phases of the molecular frontier orbital wave function.

This study evaluated the structure and reactivity of hit molecules against Mpox proteins using FMO, a technique widely used in organic chemistry to elucidate the structure and reactivity of molecules. In this case, a molecule with a low HOMO-LUMO gap energy should have a high chemical reactivity with low kinetic stability, which is considered a soft molecule. Molecules with a large FMO energy gap are more energetically stable, which means they are less chemically reactive and have higher kinetic stability compared to molecules with a smaller FMO energy gap [17]. To assess the chemical reactivity and kinetic stability of the hit molecules identified against the key Mpox proteins, the HOMO and LUMO were investigated, and the HOMO-LUMO gap energy was calculated using the equation shown in Section 2.7. Chebulic acid had HOMO and LUMO values of −0.240589 and −0.058273 (Figure 15A), respectively. The calculated HOMO-LUMO gap energy of chebulic acid is 0.182316 eV (Figure 15A and Table 6). Likewise, chlorogenic acid has HOMO and LUMO values of −0.224785 and −0.078030 (Figure 15B), respectively. The calculated HOMO-LUMO gap energy of chlorogenic acid is 0.1468 (Figure 15B and Table 6). The lowest HOMO-LUMO gap energy for these molecules implies that they have a lower kinetic stability and higher chemical reactivity.

### 3.9. Binding Free Energy Calculation

**Figure 16:**
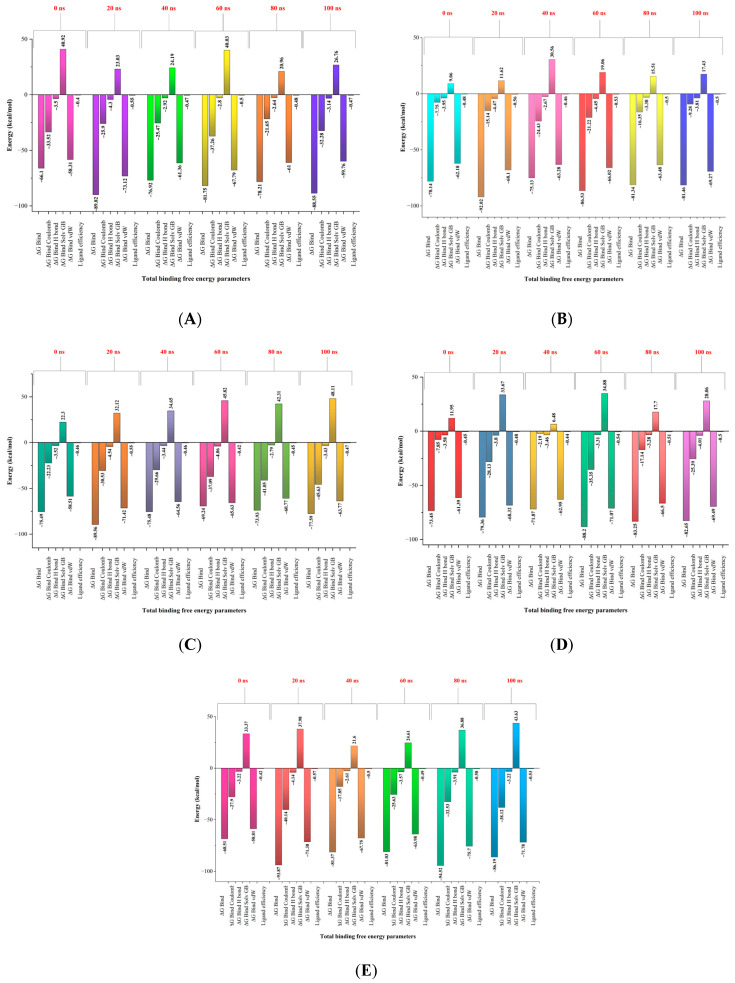
Total binding free energies of hit molecules at every 20 ns for 100 ns: (**A**) Chlorogenic acid; (**B**) Chebulic acid; (**C**) Rosmarinic acid; (**D**) Citric acid; and (**E**) Tecovirimat.

This method is highly effective in drug design research, particularly for forecasting the free binding energies associated with protein-ligand interactions. Compared to other molecular docking scoring systems, this method is more accurate, and requires less computer work than alchemical free energy methods [27,58]. The Prime MM/GBSA algorithm was used to forecast the free binding energies of ligand-protein complexes (hit molecules from the polyherbal formulation and the Mpox protein E5). The binding-free energy calculation revealed that chlorogenic acid, chebulic acid, rosmarinic acid, and citric acid had good energy values at 100 ns, which were analyzed every 20 ns until 100 ns (Figure 16A–E). The free energy parameters for binding are shown in Figure 16A–E. These include ΔG-bind, G-bind Coulomb, ΔG-bind H-bond, H-bond Solv GB, ΔG-bind VdW, and ligand efficiency. MM-GBSA analysis revealed that the binding energies of these compounds remained relatively stable under thermal conditions. This analysis revealed that the thermostatic settings had no effect on the binding affinities of the ligand-protein complexes (Figure 16A–E). This suggests that these compounds bind strongly to E5 of the Mpox virus.

### 3.10. Toxicity Profiles of Hit Molecules

**Figure 17:**
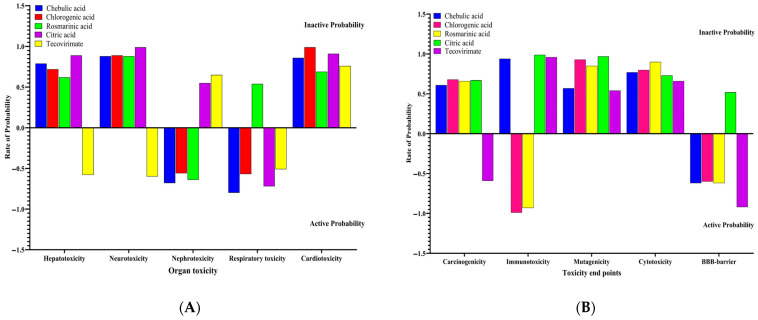
(**A**,**B**) Probability rate for organ toxicity and toxicity end points of hit molecules.

In this research, two criteria such as organ toxicity and toxicity endpoints were investigated on organic acids with higher binding affinities with Mpox E5. The risk of organ toxicity and toxicity endpoints of the organic acids were assessed using their probability scores. In this case, molecules exhibiting a higher inactive probability score suggest that they possess reduced organ toxicity and lower toxicity endpoints. Similarly, a molecule with a higher active probability score indicates that the chemical tends to cause organ toxicity and end points. In the present research, the toxicity of the organic acids was tested in terms of hepatotoxicity, neurotoxicity, nephrotoxicity, respiratory toxicity, and cardiotoxicity; while the toxicity endpoints of these compounds were explored in terms of carcinogenicity, immunotoxicity, mutagenicity, cytotoxicity and blood brain barrier. This assessment reveals that chebulic acid and chlorogenic acid were probable to cause nephrotoxicity and respiratory toxicity, with the probability rates of 0.8, 0.7, 0.5, and 0.5 (Figure 17A), respectively. It’s interesting to note that chlorogenic acid has a 50/50 probability of organ toxicity, suggesting that it could either cause toxicity or not. However, tecovirimat revealed organ toxicity for hepatotoxicity, neurotoxicity, and respiratory toxicity with probability rates of 0.58, 0.59, and 0.47 (Figure 17A), respectively. Interestingly, all organic acids were found to have higher inactive probability rates for hepatoxicity, neurotoxicity, respiratory toxicity, and cardiotoxicity, with probability rates greater than 0.8, indicating that these substances are not hazardous to organs. Assessment of toxicity endpoints revealed the presence of chlorogenic acid and rosmarinic acid in immunotoxicity, with probability rates of 0.98 and 0.95 (Figure 17B), respectively. Similarly, the tecovirimat has a higher active rate for the blood-brain barrier with a rate of 0.82; while a probability rate of 0.54 in tecovirimat indicates potential carcinogenic effects as well (Figure 17B).

## 4. Conclusions

**Figure 18:**
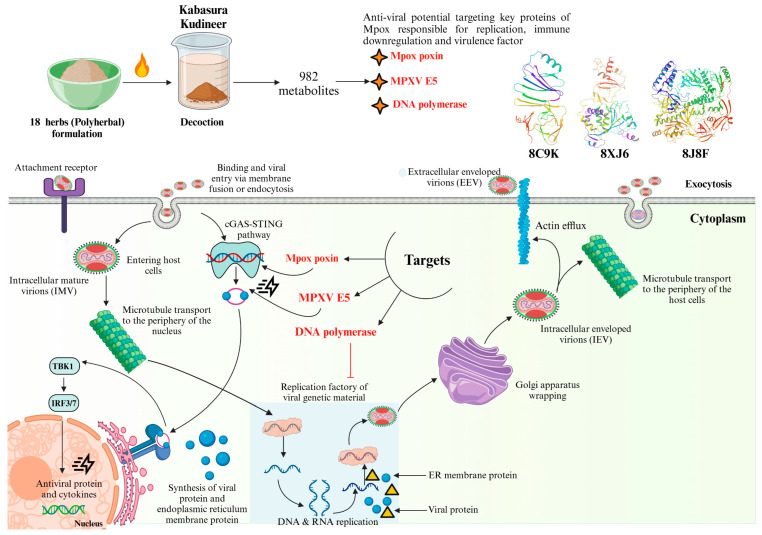
The picture depicts the mechanism of action of *Kabasura Kudineer* in reducing the complications of the Mpox virus by inhibiting viral replication, enhancing antiviral proteins and cytokines, and improving immune systems (Created with www.BioRender.com).

Currently, there is an urgent need for a novel drug candidate to mitigate the risk of this virus and its rapid transmission before it becomes a pandemic. Molecular docking studies indicated that chlorogenic acid, chebulic acid, rosmarinic acid, and citric acid, derived from the polyherbal formulation (*Kabasura Kudineer*), were the most active drug candidates for Mpox E5. Similarly, plumbagic acid, citric acid, and orientin are the most effective drugs for poxin and DNA polymerase. Among the complexes explored, MD simulations revealed that chlorogenic acid, chebulic acid, and rosmarinic acid exhibited notable stability with E5 throughout the simulation periods, exhibiting stronger rates of protein-ligand contacts than the complexes of poxin-herbs compounds (plumbagic acid and citric acid) and DNA polymerase-herbs compounds (plumbagic acid and orientin). The calculation of binding free energy showed that the hit molecules of Mpox E5 have very stable binding free energy values under thermostatic conditions for 100 ns. On the other hand, the frontier molecular orbital assessment revealed that chlorogenic acid and rosmarinic acid have lower kinetic stability and chemical reactants, implying that they cannot be stored for a longer shelf life and can easily react with other chemicals. However, the encapsulation approach can alter the kinetic stability and chemical reactivity, potentially enhancing the drug efficiency of these molecules. The present research found that all the chemical constituents of this polyherbal formulation would be effective in lowering the infection rate of the Mpox virus, especially by reducing the functions of E5, poxin, and DNA polymerase (Figure 18). However, to fully understand the mechanism of action, further in vitro and in vivo research on chlorogenic acid, chebulic acid, and rosmarinic acid against this virus are necessary. To evaluate the antiviral effects of these compounds on Mpox, the cell lines Vero E6, Vero 76, BSC-1, HEP-2, and LLC-MK2 can be used in in vitro preclinical assessment. Eventually, the present research suggests *Kabasura Kudineer* could also be effective in mitigating the complications associated with the Mpox virus. This is the first report of this formulation against the key monkeypox proteins responsible for survival and replication in the host.

## Figures and Tables

**Table 1:** Chemical constituents of polyherbal formulations that were identified as the most effective drug candidates for E5.

Name of the Compounds	IMPPAT ID	Docking Score	Glide Evdw	Glide Energy	Glide Emodel
Chlorogenic acid	IMPHY004597	−13.3289	−33.7165	−54.8584	−84.0648
Chebulic acid	IMPHY010998	−11.3933	−22.6505	−51.0801	−69.9923
Rosmarinic acid	IMPHY011844	−9.8999	−31.6954	−50.6285	−65.1073
Citric acid	IMPHY003500	−9.59471	−10.1419	−38.8322	−49.3086
Shikimic acid	IMPHY006945	−8.76481	−16.7851	−30.8403	−42.1496
d-Tartaric acid	IMPHY003999	−7.97167	−7.39175	−38.366	−48.6886
L-Rhamnose	IMPHY015056	−7.92269	−20.4093	−29.5861	−34.5808
Daidzein	IMPHY004566	−7.66304	−25.4414	−35.8084	−51.2014
Formononetin	IMPHY009035	−7.51735	−26.4555	−35.5796	−49.698
Geranic acid	IMPHY004536	−7.39299	−13.7373	−25.395	−33.2054

**Table 2:** Binding interactions of hit chemical constituents from polyherbal formulations and a prescribed drug of Mpox with Mpox E5.

S. No.	Name of the Compounds	Hydrogen Bond Interactions	Other Interactions
1.	Chlorogenic acid	ARG514 (2.01 Å), SER510 (1.84 Å), LYS509 (1.92 Å), THR507 (2.53 Å), and ALA506 (1.72 Å)	LYS509 (salt bridge), and PHE630 (pi-pi stacking)
2.	Chebulic acid	ARG514 (1.80 Å), ASP652 (1.93 Å), THR511 (2.11 Å), SER510 (2.04 Å), LYS509 (1.90 Å), and THR507 (2.06 Å)	ARG514 (bivalent: pi-cation and salt bridge), and LYS509 (salt bridge)
3.	Rosmarinic acid	ASP652 (1.91 Å), THR507 (2.59 Å), GLY508 (bivalent: 1.54 Å, 1.80 Å), SER510 (bivalent: 2.35 Å, 2.41 Å), and ASN605 (bivalent: 2.01 Å, 2.26 Å)	PHE630 (pi-pi stacking) and LYS509 (salt bridge)
4.	Citric acid	ASN605 (2.01 Å), THR505 (2.35 Å), ALA506 (1.69 Å), THR507 (2.03 Å), GLY508 (1.91 Å), LYS509 (1.77 Å), SER510 (bivalent: 2.11 Å, 2.10 Å)	LYS509 (bivalent: salt bridge)
5.	Tecovirimat	ARG514 (bivalent: 2.66 Å, 1.77 Å), and GLY508 (2.49 Å)	PHE630 (pi-pi stacking)

**Table 3:** Calculated residue-wise energy decomposition analysis from MM-GBSA of top hit molecules-Mpox E5 complexes on last 100 ns simulation trajectory.

S. No.	Name of the Compounds	Key Residues	ΔG Bind	ΔG Bind Coulomb	ΔG Bind Solvation	ΔG Bind vdW
1.	Chlorogenic acid	THR507	0.03	−0.24	0.29	−0.02
		GLY508	0.07	−0.19	0.28	−0.02
		SER510	0.03	−0.29	0.56	−0.19
		THR511	−0.11	−0.23	0.15	−0.03
		ASP652	0.01	0.83	−0.81	0
2.	Chebulic acid	GLY508	−0.17	−0.12	−0.02	−0.03
		THR507	−0.92	−0.49	0.5	−0.68
		SER510	−1.19	−9.08	8.91	−0.66
		THR511	−6.33	−3.06	1.76	−3.87
		AGR514	−3.6	0.26	−0.88	−2.36
3.	Citric acid	ASN605	−0.15	−0.3	0.18	−0.02
		ALA506	−0.17	−0.31	0.18	−0.03
		THR507	0.15	−0.7	0.96	−0.1
		LYS509	−0.65	−7.9	8	−0.74
		SER510	0.02	0.01	0.01	0
		ASP603	0.06	−0.19	0.28	−0.02
4.	Rosmarinic acid	ALA506	0	0.05	−0.04	−0.01
		GLY508	0	−0.22	0.24	−0.02
		ASP652	0.02	0.59	−0.56	0

**Table 4:** Chemical constituents of polyherbal formulations that identified as the most effective drug candidates for poxin.

Name of the Compounds	IMPPAT ID	Docking Score	Glide Evdw	Glide Energy	Glide Emodel
Caffeic acid	IMPHY007396	−8.49023	−14.6795	−20.8696	−17.7572
Citric acid	IMPHY003500	−6.80386	−5.57202	−19.9455	−19.814
Plumbagic acid	IMPHY007327	−5.91719	−14.8537	−18.1409	−17.2802
Palmitic acid	IMPHY011933	−5.83839	−12.7641	−23.2243	−25.9355
d-Tartaric acid	IMPHY003999	−5.82919	−5.20123	−17.5407	−17.7917
Esculetin	IMPHY011518	−5.33922	−13.7908	−19.9468	−25.8948
Gallic acid	IMPHY012021	−5.26569	−9.301	−16.1358	−21.3437
Hexanoic acid	IMPHY007354	−5.17643	−6.53937	−11.5305	−13.0665
Costic acid	IMPHY007157	−5.14485	−16.0833	−18.5526	−20.8173
3-Hydroxyflavone	IMPHY000011	−4.71851	−18.2404	−22.0236	−24.973

**Table 5:** Chemical constituents of polyherbal formulations identified as the most effective drug candidates for DNA polymerase.

Name of the Compounds	IMPPAT ID	Docking Score	Glide Evdw	Glide Energy	Glide Emodel
Plumbagic acid	IMPHY005455	−7.57867	−5.97257	−27.1799	−34.553
Delphinidin	IMPHY012050	−7.55301	−27.7059	−51.6504	−71.5141
D-Glucose	IMPHY014893	−7.54528	−11.1257	−29.7152	−33.1126
(−)-Epicatechin	IMPHY014908	−7.21082	−18.711	−44.2525	−55.5263
Quercitol	IMPHY011805	−7.07336	−9.88762	−31.0983	−35.4338
D-Galactose	IMPHY007396	−6.82773	−18.0707	−32.6402	−40.5891
L-Rhamnose	IMPHY015056	−6.40755	−15.1972	−28.6819	−33.426
Gallic acid	IMPHY012021	−6.31696	−11.2991	−20.4942	−24.3306
Orientin	IMPHY007124	−6.17408	−22.4098	−46.8336	−59.4742
D-Xylose	IMPHY015116	−6.16501	−10.9548	−28.158	−36.1138

**Table 6:** Frontier molecular orbital features of chlorogenic acid and chebulic acid.

Name of the Compounds	E_HOMO_	E_LUMO_	Gap Energy (eV)	Ionization Potential (P)	Electron Affinity (A)	Electrophilicity Index (ω)	Hardness (eV)
Chlorogenic acid	−0.2247	−0.0780	0.1468	0.2248	0.1468	0.1468	0.0734
Chebulic acid	−0.2405	0.0582	0.1823	0.2406	0.1823	0.1824	0.0912

## Data Availability

All the data are enclosed with this manuscript.
